# Mutually exclusive epigenetic modification on *SIX6* with hypermethylation for precancerous stage and metastasis emergence tracing

**DOI:** 10.1038/s41392-022-01026-7

**Published:** 2022-07-06

**Authors:** Shihua Dong, Zhicong Yang, Peng Xu, Wanxiang Zheng, Baolong Zhang, Fangqiu Fu, Zhanrui Mao, Jianlin Yuan, Haiquan Chen, Wenqiang Yu

**Affiliations:** 1 Shanghai Epiprobe Biotechnology Co., Ltd, Shanghai, China; 2 Shanghai Public Health Clinical Center & Department of General Surgery, Huashan Hospital & Cancer Metastasis Institute & Institutes of Biomedical Sciences, Shanghai Medical College, Fudan University, Shanghai, China; 3 Department of Urology, Xijing Hospital, Air Force Medical University, Xi’an, Shaanxi China; 4 Department of Thoracic Surgery, Fudan University Shanghai Cancer Center & Institute of Thoracic Oncology & State Key Laboratory of Genetic Engineering, Fudan University, Shanghai, China

**Keywords:** Tumour biomarkers, Predictive markers

**Dear Editor**,

Aberrant DNA methylation gets involved in cancer initiation, progression, and recurrence, which in turn makes it an ideal cancer biomarker. Various methylation markers or their panels have been developed in diverse cancer types. However, the model-constructing based marker mining strategy and incompatibility of application have greatly impeded their ways to clinic. Thus, single methylation marker applicable to all/most cancer types and multiple clinical scenarios is desperately needed. The hope came from the unexpected observation that *HIST1H4F* was universally hypermethylated in all 17 cancer types; thus, we raised the concept of “Universal Cancer Only Marker (UCOM)” and established a paradigm for discovery and clinical application of UCOM.^1^ Recently, a novel UCOM, hypermethylated *PCDHGB7*, was identified and found to advance cervical cancer (CC) screening to the precancerous stage.^2^ During the screening of UCOM, we discerned a bunch of cancer cell-differentially methylated regions.^1^ Among them, *sine oculis* (SIX) homeobox family of transcription factors, which were found to function as tumorigenesis regulator by promoting epithelial-to-mesenchymal transition and metastasis recently in addition to their traditional roles in tissue formation and organogenesis,^3^ sparked our special attention. Herein, we interrogate whether *SIX6* methylation could serve as a novel UCOM and its potential applications.

Firstly, WGBS data of 78 samples (Supplementary Table S1) revealed *SIX6* methylation level in normals (<10%) was significantly lower than in cancers (>50% in 13/15 samples) (Supplementary Fig. S1a). To systematically investigate *SIX6* methylation status, we resorted to 7010 samples across 15 cancer types from TCGA datasets (Supplementary Table S2). It demonstrated that *SIX6* was universally hypermethylated in cancer samples compared with the normals (Supplementary Fig. S1b). Likewise, we collected 678 clinical samples across ten common cancer types (Supplementary Table S3) and confirmed *SIX6* hypermethylation in all available cancer types (Fig. 1a). These data strongly suggested hypermethylated *SIX6* as a novel UCOM.

**Fig. 1:**
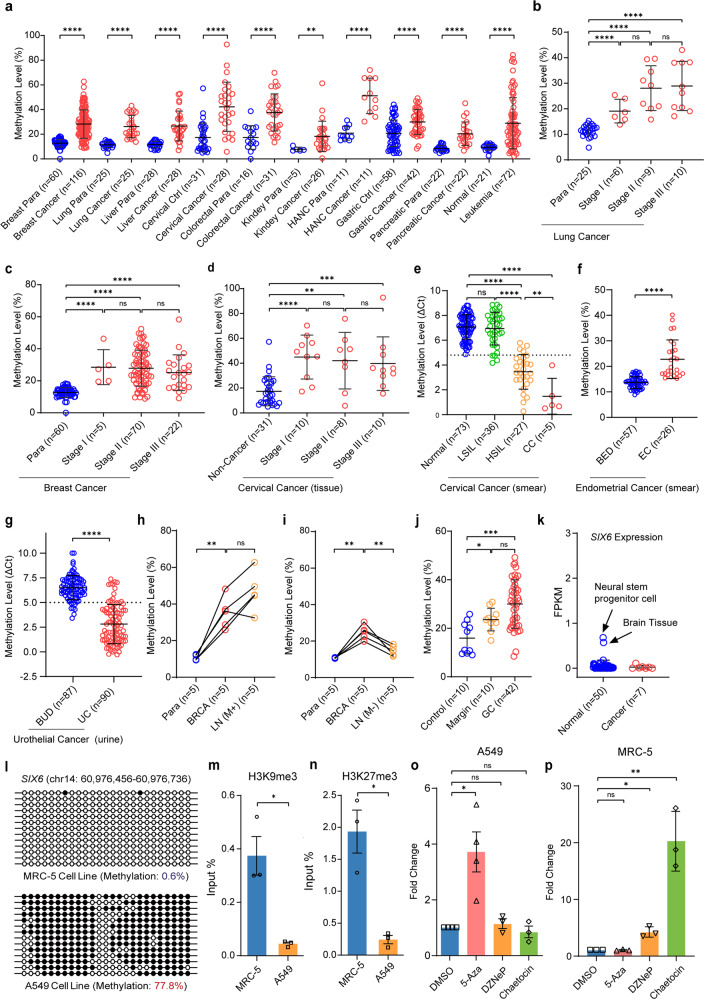
Hypermethylated *SIX6* as a novel UCOM for precancerous stage and metastasis emergence tracing. **a** *SIX6* methylation level in 678 clinical samples across ten common types of cancer and normal tissues. HANC, head and neck cancer. **b**–**d** *SIX6* methylation level in para-cancer, stage I, stage II, and stage III tissues of lung cancer (**b**), breast cancer (**c**), and cervical cancer (**d**). **e**–**g** *SIX6* methylation level in the pathological progression of cervical cancer (**e**) endometrial cancer (**f**), and urothelial cancer (**g**). LSIL: low-grade squamous intraepithelial lesion; HSIL: high-grade squamous intraepithelial lesion; CC: cervical cancer; BED, benign endometrial diseases; EC, endometrial cancer; BUD, benign urothelial diseases; UC, urothelial cancer. Note that methylation detection in cervical smear (**e**) and urine (**g**) was measured by MSRE-qPCR, and the lower ΔCt indicates the higher methylation level. **h**, **i** *SIX6* methylation level in para-cancer, breast invasive carcinoma (BRCA), lymph node metastasis-positive samples (LN(M+)), and lymph node metastasis-negative samples (LN(M−)), respectively. **j** *SIX6* methylation level in para-cancer, surgical margin, and gastric cancer. **k** Gene expression analysis of *SIX6* in normal and cancer samples from ENCODE datasets. **l** *SIX6* methylation level was measured in A549 and MRC-5 by Sanger sequencing from bisulfite-treated DNA. Each circle represents a single cytosine. The solid circle (●) represents methylated cytosine, and the open circle (○) represents unmethylated cytosine. **m**, **n** Enrichment of repressive histone markers H3K9me3 (**m**) and H3K27me3 (**n**) modifications was measured by ChIP-qPCR in A549 and MRC-5 cell lines. **o**, **p** Gene expression analysis of *SIX6* in A549 (**o**) and MRC-5 (**p**) cell lines after the treatment of DNA methylation inhibitor 5-Aza, H3K27me3 inhibitor DZNep, and H3K9me3 inhibitor Chaetocin. Error bar represents upper quartile, lower quartile, and median. *P* values were calculated using the two-tailed nonparametric Mann-Whitney test (**a**–**k**) and Paired t-test (**m**–**p**) by GraphPad Prism 7.0 software. ns not significant; *P < 0.05, **P < 0.01, ***P < 0.001, ****P < 0.0001

Early screening is one of the most economical and effective ways to reduce cancer mortality. The in-depth analysis of TCGA datasets unveiled that *SIX6* hypermethylation occurred in the early stage of 12 cancer types (Supplementary Fig. S2a–l), which was further validated in the two most dominant cancer types, lung cancer and breast cancer, in clinical samples (Fig. 1b, c). CC is the first cancer type that was declared to be eliminated by 2030, the incidence and mortality of which could be reduced by early screening. It showcased that *SIX6* already exhibited hypermethylation in stage I of CC (Fig. 1d). Moreover, compared to low-grade squamous intraepithelial lesion (LSIL), *SIX6* was significantly hypermethylated in high-grade squamous intraepithelial lesion (HSIL) and CC (Supplementary Fig. S3a). To make it a more user-friendly approach, we measured *SIX6* methylation status by MSRE-qPCR in cervical smear, a non-invasive sample for CC screening, and the higher methylation was also observed in HSIL and CC (Fig. 1e). Methylation detection in cervical smear could also be applied in endometrial cancer (EC) screening,^4^ which was validated by *SIX6* hypermethylation in EC compared with benign endometrial diseases (BED) (Fig. 1f). Another ideal material for cancer screening is urine, in which *SIX6* methylation status enables the significant discrimination between urothelial cancer (UC) and benign urothelial diseases (BUD) (Fig. 1g). The ROC curve showed AUC of CC, EC, UC were 0.99, 0.94, 0.93, respectively (Supplementary Fig. S3b) with excellent sensitivity and specificity (Supplementary Fig. S3c). The similar phenomenon was observed in gastric cancer, and *SIX6* was significantly hypermethylated in high-gastric-cancer-risk (HGCR, including atrophy gastritis and HSIL) and gastric cancer, compared with low-gastric-cancer-risk (LGCR, including chronic non-atrophic gastritis, gastricism, and superficial gastritis) (Supplementary Fig. S3d). Additionally, compared to normals, chronic leukemia harbored higher *SIX6* methylation level, which increased progressively in acute leukemia (Supplementary Fig. S3e). These data strongly support *SIX6* hypermethylation is an early event in tumor progression, especially when normal cells transform into precancerous cells.

Once diagnosed with cancer, metastasis is a critical factor that affects treatment strategy selection and prognosis. Therefore, we interrogated whether *SIX6* hypermethylation could function as a molecular biomarker for tracing cancer metastasis by detecting its level in the lymph node of cancer at the potential metastatic stage. Like in corresponding breast cancer tissues, *SIX6* methylation level in lymph node with metastasis-positive samples was also in a hypermethylated state (Fig. 1h), while it was significantly lower in lymph node metastasis-negative samples (Fig. 1i). These data suggested that *SIX6* methylation detection may serve as a predictor for lymph node metastasis in breast cancer. Generally, surgical margins were determined by pathological analysis during operation. The methylation level of *SIX6* from surgical margins were higher than distal surgical ones (Fig. 1j). This data not only indicates the pathologist experience-based pathology needs improvement, but more importantly, *SIX6* hypermethylation could serve as an indicator for precision surgery and prognosis prediction in a time-and-cost-effective manner.

Considering DNA methylation is a well-established gene expression regulator, we analyzed *SIX6* gene expression in seven cancer samples compared to 50 normal tissues from ENCODE (Supplementary Table S4). As expected, *SIX6* was silenced in cancer samples. Surprisingly, *SIX6* was also not expressed in most normal samples, except minor expression (FPKM ≤ 1.0) in brain tissue and neural stem progenitor cells (Fig. 1k), which was supported by whole body expression profile of *SIX6.*^5^ That was also the case in lung cancer cell line A549 and normal lung cell line MRC-5 (Supplementary Fig. S4a). It was noteworthy that *SIX6* was hypermethylated in A549, but hypomethylated in MRC-5 (Fig. 1l). These data hinted that hypermethylation may account for *SIX6* silencing in cancer samples and there exist unrecognized mechanisms in normal samples. Repressive histone modifications H3K27me3 and H3K9me3 were significantly higher in MRC-5 compared with A549 (Fig. 1m, n), but not active markers H3K4me1, H3K4me3, and H3K27ac (Supplementary Fig. S4b–d). Except for lung cancer, *SIX6* was also silenced in bladder carcinoma cells T24 and normal bladder cell line CCC-HB-2 (Supplementary Fig. S4e). Similarly, significant hypermethylation and H3K27me3 enrichment were observed in T24 and CCC-HB-2, respectively (Supplementary Fig. S4f, g). The epigenetic switching was further evidenced by the similar observations in prostate and breast cancer (Supplementary Fig. S4h).

To further validate this epigenetic modification switch-based expression silence, we interfered cells with inhibitors. DNA methylation inhibitor 5-azacitidine (5-Aza) treatment efficiently reduced *SIX6* methylation level in A549 (Supplementary Fig. S4i). Meanwhile, H3K27me3 inhibitor 3-deazaneplanocin A (DZNep) and H3K9me3 inhibitor Chaetocin significantly decreased H3K27me3 and H3K9me3 level in MRC-5, respectively (Supplementary Fig. S4j, k). Importantly, 5-Aza treatment upregulated the expression of *SIX6* in A549, while DZNep or Chaetocin treatment increased *SIX6* expression in MRC-5 (Fig. 1o, p). Collectively, these results imply that in normal cells *SIX6* silencing is mediated by repressive histone modifications, such as H3K27me3 and H3K9me3, while DNA methylation replaces them to mediate the silencing of *SIX6* in cancers (Supplementary Fig. S4l). It will be of great interest to investigate the mutually exclusive epigenetic modification induced distinctive silencing phenomena during tumorigenesis and progression.

In sum, this study identified hypermethylated *SIX6* as a novel UCOM for early cancer screening, which can also be used as an indicating marker for precancerous stage and metastasis emergence tracing. Moreover, epigenetic silencing of *SIX6* was regulated by hypermethylation and repressive histone modifications in cancer and normal tissues in a mutually exclusive pattern, respectively. Our study reveals that hypermethylated *SIX6* acts as a UCOM, which provides new insights into the unexplored functions of *SIX6*.

## Supplementary information

Supplementary

TableS1-S5

## Data Availability

The data presented in this study have been included in the article and Supplementary Materials. Further requests could be made to the corresponding authors.
