# Supplementary material for: Mutually exclusive epigenetic modification on SIX6 with hypermethylation for precancerous stage and metastasis emergence tracing
Source: Signal Transduct Target Ther. 2022 Jul 6;7:208. doi: 10.1038/s41392-022-01026-7 (PMC9256699; doi:10.1038/s41392-022-01026-7)
Supplement: Supplementary file 1 — Supplementary [file 41392_2022_1026_MOESM1_ESM.docx]

Supplemental Materials

**Mutually exclusive epigenetic modification on *SIX6* with hypermethylation for** **precancerous stage and metastasis emergence tracing**

Shihua Dong^1^, Zhicong Yang^2^, Peng Xu^1^, Wanxiang Zheng^3^, Baolong Zhang^2^, Fangqiu Fu^4^, Zhanrui Mao^1^, Jianlin Yuan^3^*, Haiquan Chen^4^*, Wenqiang Yu^2^*

^1^ Shanghai Epiprobe Biotechnology Co., Ltd, Shanghai, China.

^2^ Shanghai Public Health Clinical Center & Department of General Surgery, Huashan Hospital & Cancer Metastasis Institute & Institutes of Biomedical Sciences, Shanghai Medical College, Fudan University, Shanghai, China.

^3^ Department of Urology, Xijing Hospital, Air Force Medical University, Xi’an, Shaan Xi, China.

^4^ Department of Thoracic Surgery, Fudan University Shanghai Cancer Center & Institute of Thoracic Oncology & State Key Laboratory of Genetic Engineering, Fudan University, Shanghai, China.

These authors contributed equally: Shihua Dong, Zhicong Yang, Peng Xu, Wanxiang Zheng, and Baolong Zhang.

*Corresponding authors:
Wenqiang Yu ([wenqiangyu@fudan.edu.cn](mailto:wenqiangyu@fudan.edu.cn))

Haiquan Chen ([hqchen1@yahoo.com](mailto:hqchen1@yahoo.com))

Jianlin Yuan ([jianliny@fmmu.edu.cn](mailto:jianliny@fmmu.edu.cn))

**Supplementary information includes:**Materials and Methods

Supplementary Figures S1 – S4

Supplementary Table S1 – S5

**Materials and Methods**

**WGBS dataset analysis**

A total of 78 whole-genome bisulfite sequencing (WGBS) datasets, including 15 cancer and 63 normal samples, of which the FASTQ reads data or calculated BED (Browser Extensible Data) files were downloaded from Encyclopedia of DNA Elements (ENCODE), Sequence Read Archive (SRA) or Gene Expression Omnibus (GEO) database. The sources of data were listed in Supplementary Table S1. WGBS data were analyzed by BSMAP software, and methylation value for each CpG site was calculated by covered reads C/(C+T). CpG sites covered above five reads within *SIX6* gene promoter region (TSS±500bp) were selected for DNA methylation calculation. Final methylation value for each sample was calculated by the average of each qualified CpG site.

**TCGA dataset analysis**

The Illumina 450K methylation array data from the TCGA database were downloaded from the UCSC Xena browser (<https://xenabrowser.net/>). A total of 7010 clinical cancer samples of 15 cancer types were enrolled in the analysis. The absolute methylation values were calculated from the β values of 450K methylation array [methylation value = (β value + 0.5) ×100%]. The final methylation values were calculated by the average of three probes (cg06785999, cg14186066, cg19456540) embedded in *SIX6* promoter. All samples used from the TCGA project and their corresponding methylation levels were listed in Supplementary Table S2. KICA, kidney cancer, includes kidney chromophobe (KICH), kidney renal clear cell carcinoma (KIRC), and kidney renal papillary cell carcinoma (KIRP) three cohorts in the TCGA database. LUNG, lung cancer, includes lung adenocarcinoma (LUAD) and lung squamous cell carcinoma (LUSC) two cohorts.

**Clinical samples**

A total of 1079 clinical samples across 12 types of cancer were collected from Xijing Hospital of Air Force Medical University and Fudan University Shanghai Cancer Center, including breast cancer (cancer: n = 116; unpaired para-cancer: n = 60), lung cancer (cancer: n = 25; paired para-cancer: n = 25), liver cancer (cancer: n = 28; paired para-cancer: n = 28), cervical cancer (cancer tissue: n = 28; unpaired non-cancer tissue: n = 31; cancer smear: n = 5; unpaired non-cancer smear: n = 136), colorectal cancer (cancer: n = 31, unpaired non-cancer: n = 16), endometrial cancer (cancer smear: n = 26; benign endometrial diseases smear: n = 57); kidney cancer (cancer: n = 26; unpaired para-cancer: n = 5), head and neck cancer (cancer: n = 11; paired para-cancer: n = 11), gastric cancer (cancer: n = 42; unpaired non-cancer: n = 58), pancreatic cancer (cancer: n = 22; paired para-cancer: n = 22), and leukemia (cancer: n = 72; normal: n = 21); urothelial cancer (cancer urine: n=90; benign urothelial diseases urine: n = 87). The detailed DNA methylation levels of 1079 clinical samples were illustrated in Supplementary Table S3. Note that gastric cancer samples were taken from tumor foci, surgical margin samples were taken from the surgical margin which closest to the tumor (usually 2-5 cm), and control samples were taken from the most distal surgical margin from tumor or non-cancer patients.

**Cell lines**

A549 (adenocarcinomic human alveolar basal epithelial cells), MRC-5 (human fetal lung fibroblast cells), T24 (human urinary bladder carcinoma cells), and CCC-HB-2 (human urinary bladder cells) were from our laboratory (Wenqiang Yu’s lab at Fudan University). CCC-HB-2 was cultured in DMEM/High Glucose medium supplemented with 1% Penicillin-Streptomycin and 20% FBS, while the other three with 10% FBS, at 37°C under 5% CO_2_.

**DNA extraction, bisulfite-PCR pyrosequencing, and MSRE-qPCR**

DNA extraction from frozen tissue, smear, urine or FFPE samples was conducted using QIAGEN DNA extraction kit (Qiagen, 51306 and 56404). For bisulfite-PCR pyrosequencing, 200~500ng of gDNA was bisulfite converted with EZ DNA Methylation-Gold Kit (ZYMO Research, D5006), and the recovered bisulfite-treated DNA was used as the subsequent PCR template. Six CpG sites in *SIX6* genomic locus (Chr14: 60,976,454-60,976,503, GRCh38/hg38) were used. The PCR program was set at 98°C 30s for pre-denaturation; 98°C 10s, 58°C 30s, 72°C 30s for 45-cycle amplification, and 72°C 3min for final elongation. The amplified PCR products were confirmed by 2% agarose gel electrophoresis. The pyrosequencing assay was performed on a PyroMark instrument (Qiagen, Q96 ID). The methylation level of *SIX6* promoter region was calculated as the average value of six CpG sites.

MSRE-qPCR was also used for methylation detection, which was described previously.^1^ Briefly, the target genomic region harbors several cutting sites. Mechanically, if the CpG dinucleotide within restriction enzymes cutting site was methylated, it cannot be digested; if not, it can be digested but cannot be amplified and detected by subsequent qPCR. The *GAPDH* gene region absent of any cutting sites was selected for normalization. For each digestion reaction, 100 ng of gDNA was taken as input, and endonucleases were added to make the final volume 25μl, followed by digestion at 37°C for 30 min and heat inactivation at 95°C for 5 min. The subsequent dual real-time PCR was performed on 7500 Real-Time PCR System (Life Technologies) with a program as follows: initiation at 95°C for 10 min; then 45 cycles of 94°C for 20 s, 60°C for 60 s. The DNA methylation level for each sample was evaluated by ΔCt = Ct_*_SIX6_* – Ct_*_GAPDH_*.

**ChIP-qPCR and RT-qPCR**

ChIP was carried out in A549, MRC-5, CCC-HB-2, and T24 cell lines according to the standard protocol. Briefly, approximate 300μg fixed chromatin and 5μg antibody, including H3K9me3 (Abcam, ab8898) and H3K27me3 (Abcam, ab6002) were used for each ChIP pull-down. Total RNA was extracted with TRIzol (Ambion), and 1μg total RNA was used for reverse transcription with PrimeScript™ RT reagent Kit (TaKaRa) to get the cDNA. ChIP-DNA and cDNA was detected with SuperReal PreMix Plus (SYBR Green) (Tiangen Bio) in LightCycler 480 Real-time PCR system (Roche). GAPDH was used as an internal control for mRNA. The primer sequences for detection of *GAPDH*, specific histone-enriched regions, and *SIX6* were listed in Supplementary Table S5.

**Drug treatment**

MRC-5 and A549 cell lines were treated with 1.0μM 5-azacitidine (5-Aza) (Sigma, A3656) for 7 days, 1.0μM DZNep (Selleck, S7120) for 3 days, or 100nM Chaetocin (Selleck, S8068) for 24h.

**Statistical analysis**

Statistical analysis was conducted by GraphPad Prism 7.0 software. The differences between two independent groups of large samples (**Fig. 1a-k**) were compared using two-tailed nonparametric Mann-Whitney test. Comparisons between two groups of small samples (**Fig. 1m-p**) were performed with Student’s t-test. *P* < 0.05 was considered as statistical significance.

**Reference:**

1 Dong, S. *et al.* Hypermethylated PCDHGB7 as a universal cancer only marker and its application in early cervical cancer screening. *Clin Transl Med*. **11**, e457, (2021).


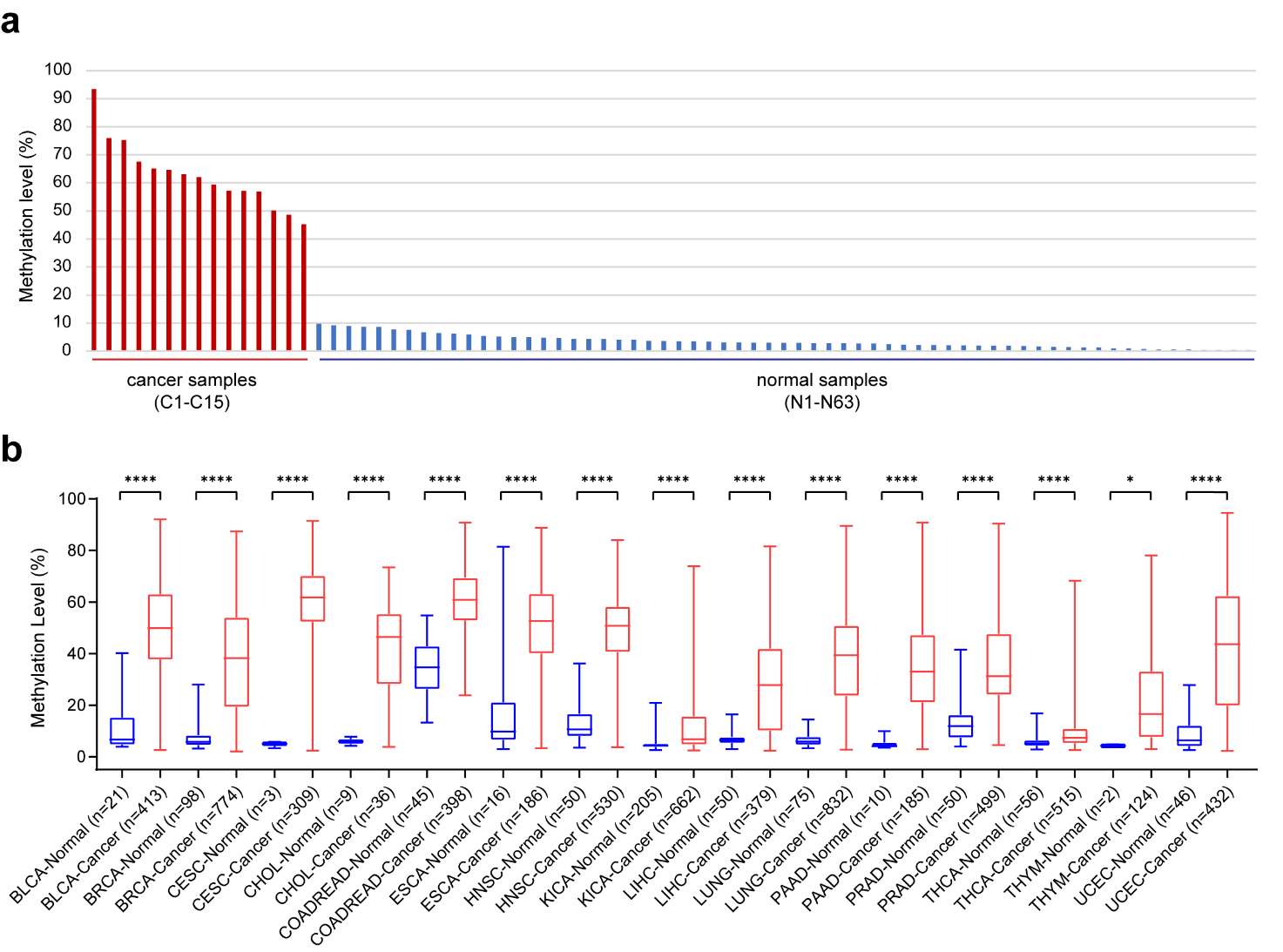


**Supplementary Fig S1.** Hypermethylated *SIX6* was identified as a Universal Cancer Only Marker (UCOM). **a** *SIX6* methylation level in 63 normal (N1~N63) and 15 cancer samples (C1~C15) from WGBS data. **b** Pronounced *SIX6* hypermethylation was confirmed in 15 cancer types from the TCGA database. BLCA, bladder urothelial carcinoma; BRCA, breast invasive carcinoma; CESC, cervical squamous cell carcinoma and endocervical adenocarcinoma; CHOL, cholangiocarcinoma; COADREAD, colon adenocarcinoma and rectum adenocarcinoma; ESCA, esophageal carcinoma; HNSC, head and neck squamous cell carcinoma; KICA, kidney cancer, include kidney chromophobe (KICH), kidney renal clear cell carcinoma (KIRC), and kidney renal papillary cell carcinoma (KIRP); LIHC, liver hepatocellular carcinoma; LUNG, lung cancer, includes lung adenocarcinoma (LUAD) and lung squamous cell carcinoma (LUSC); PAAD, pancreatic adenocarcinoma; PRAD, prostate adenocarcinoma; THCA, thyroid carcinoma; THYM, thymoma; UCEC, uterine corpus endometrial carcinoma. The boxes represent the median ± 1 quartile, with the whiskers extending from the hinge to the smallest or largest value. Error bar represents upper quartile, lower quartile, and median. *P* values were calculated using the two-tailed nonparametric Mann-Whitney test by GraphPad Prism 7.0 software. *, *P* < 0.05; ****, *P* < 0.0001.


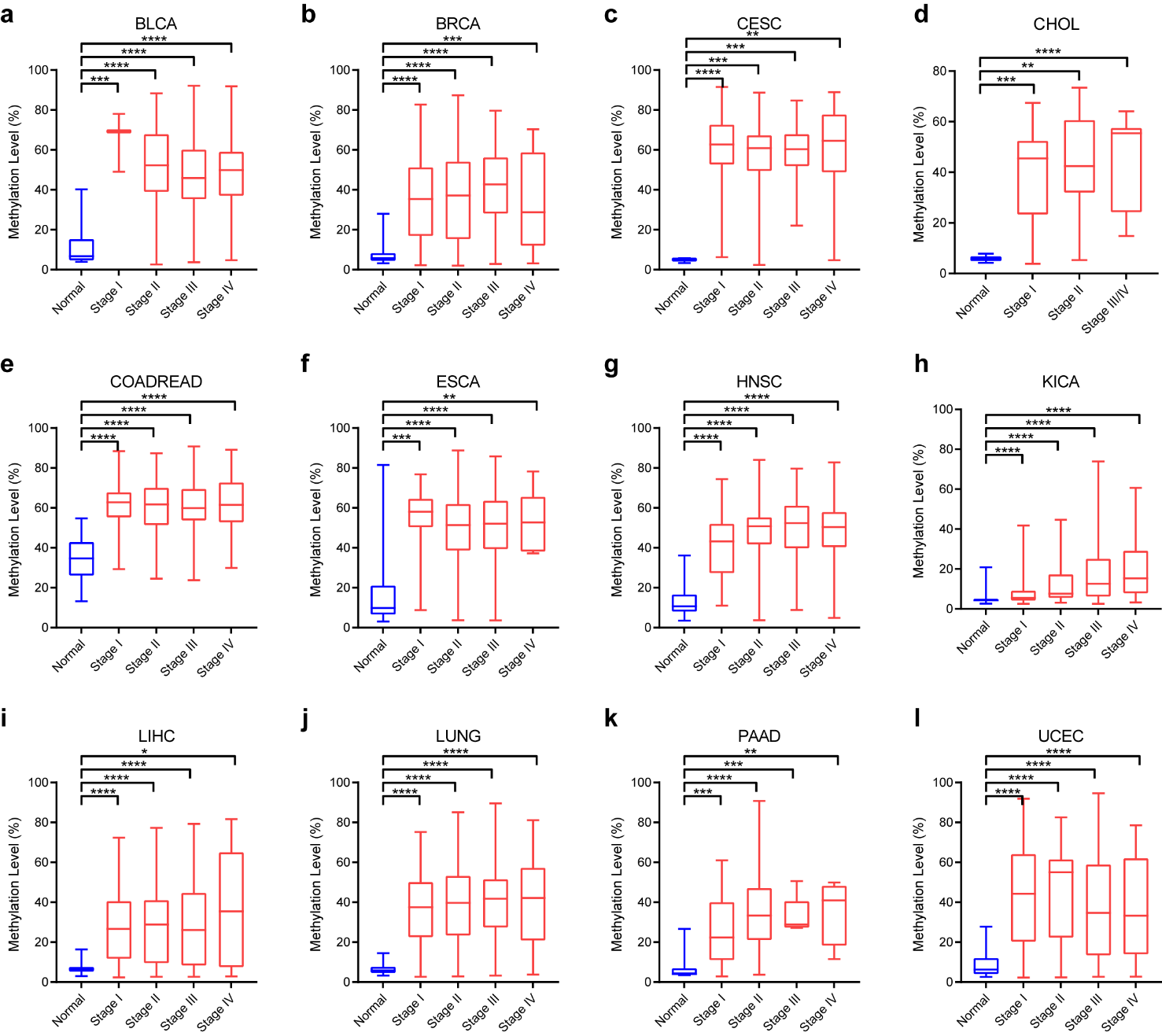


**Supplementary Fig S2.** *SIX6* already exhibited DNA hypermethylation in stage I of cancer progression. **a-l,** *SIX6* methylation level in normal and different stages of cancer samples from the TCGA database. BLCA, bladder urothelial carcinoma; BRCA, breast invasive carcinoma; CESC, cervical squamous cell carcinoma and endocervical adenocarcinoma; CHOL, cholangiocarcinoma; COADREAD, colon adenocarcinoma and rectum adenocarcinoma; ESCA, esophageal carcinoma; HNSC, head and neck squamous cell carcinoma; KICA, kidney cancer, includes kidney chromophobe (KICH), kidney renal clear cell carcinoma (KIRC) and kidney renal papillary cell carcinoma (KIRP); LIHC, liver hepatocellular carcinoma; LUNG, lung adenocarcinoma (LUAD) and lung squamous cell carcinoma (LUSC); PAAD, pancreatic adenocarcinoma; UCEC, uterine corpus endometrial carcinoma. The boxes represent the median ± 1 quartile, with the whiskers extending from the hinge to the smallest or largest value. *P* values were calculated using the two-tailed nonparametric Mann-Whitney test by GraphPad Prism 7.0 software. *, *P* < 0.05; **, *P* < 0.01; ***, *P* < 0.001; ****, *P* < 0.0001.


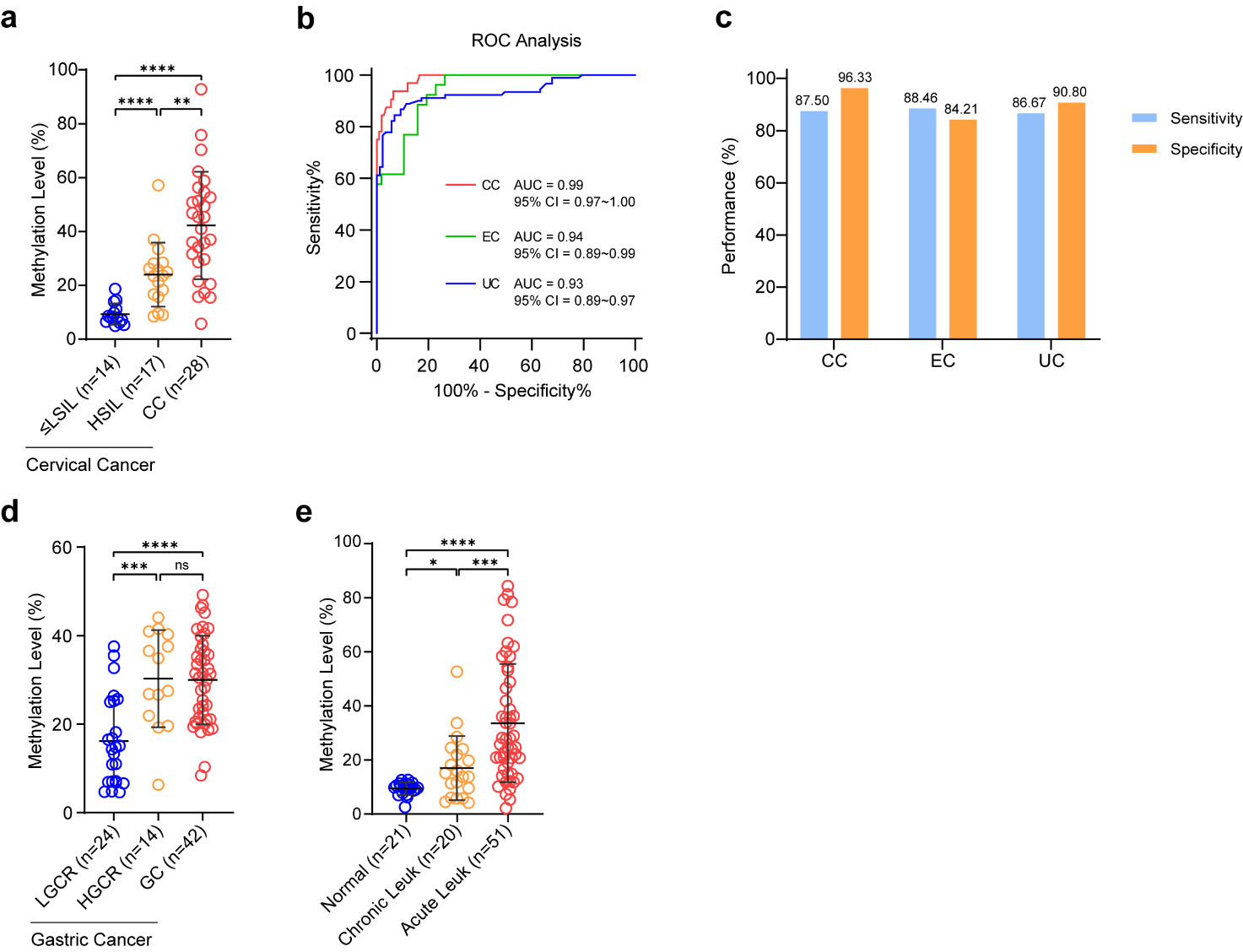


**Supplementary Fig S3.** *SIX6* hypermethylation functioned as an early screening marker. **a** *SIX6* methylation level in the pathological progression of cervical cancer. ≤LSIL includes normal and LSIL samples. **b** The ROC curve showed the AUC of CC, EC, UC were 0.99, 0.94, 0.93, respectively. **c** The sensitivity and specificity of *SIX6* methylation in CC, EC, and UC detection. **d** *SIX6* methylation level in the pathological progression of gastric cancer. LGCR: low gastric cancer risk; HGCR: high gastric cancer risk; GC: gastric cancer. **e** *SIX6* methylation level was detected in chronic leukemia and acute leukemia. *P* values were calculated using the two-tailed nonparametric Mann-Whitney test by GraphPad Prism 7.0 software. ns, not significant; *, *P* < 0.05; **, *P* < 0.01; ***, *P* < 0.001; ****, *P* < 0.0001.


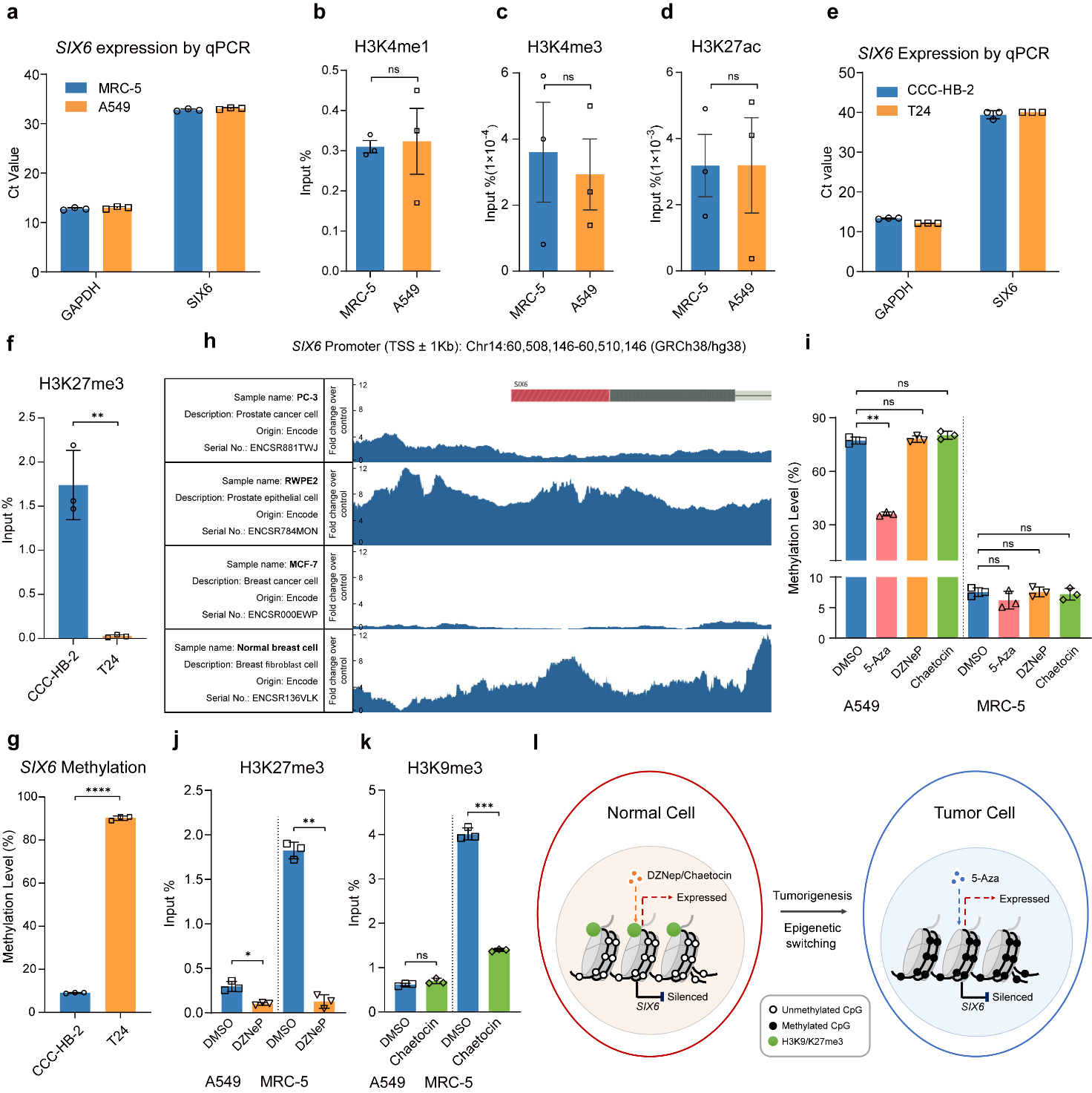


**Supplementary Fig S4.** Mutually exclusive epigenetic modification accounts for silencing of *SIX6*. **a** RT-qPCR was used to detect the expression of *SIX6* in MRC-5 and A549 cell lines. **b-d** Enrichment of active histone markers H3K4me1 (**b**), H3K4me3 (**c**), and H3K27ac (**d**) modifications was measured by ChIP-qPCR in A549 and MRC-5 cell lines. **e-g** *SIX6* expression detected by RT-qPCR (**e**), H3K27me3 enrichment measured by ChIP-qPCR (**f**), and *SIX6* methylation level measured by pyrosequencing (**g**) in CCC-HB-2 and T24 cell lines. **h** H3K27me3 enrichment in prostate cancer cell PC-3, prostate epithelial cell RWPE2, breast cancer cell MCF-7, and normal breast cell from ENCODE datasets. **i** DNA methylation detection assay in MRC-5 and A549 cell lines after the treatment of DNA methylation inhibitor 5-Aza, H3K27me3 inhibitor DZNep, and H3K9me3 inhibitor Chaetocin. **j-k** ChIP-qPCR analysis of H3K27me3 and H3K9me3 enrichment in MRC-5 and A549 cell lines after the treatment of DZNep and Chaetocin, respectively. **l** The schematic representation of the mechanism underlying mutually exclusive epigenetic modifications (MEM) surrounding *SIX6* locus account for its epigenetic silencing, and this characteristic of epigenetic switching contributes to tumorigenesis and allows hypermethylated *SIX6* to act as a potent UCOM. *P* values were calculated using the Paired t-test by GraphPad Prism 7.0 software. ns, not significant; **, *P* < 0.01; ***, *P* < 0.001; ****, *P* < 0.0001.

**Supplementary Table 1:** Detailed list of WGBS datasets.

**Supplementary Table 2:** Detailed list of analyzed TCGA data.

**Supplementary Table 3:** Detailed information of clinical samples.

**Supplementary Table 4:** *SIX6* expression in normal and cancer tissues.

**Supplementary Table 5:** Details of primers used for ChIP-qPCR and RT-qPCR.
